# A rare mammoth nevus lipomatosus superficialis: Health care avoidance in patients with distressing cosmetic conditions

**DOI:** 10.1016/j.jdcr.2025.02.047

**Published:** 2025-04-09

**Authors:** Claire Rose Kissinger, Aspen Trautz, Blake Elizabeth Brooks

**Affiliations:** a Lewis Katz School of Medicine at Temple University/St. Luke’s University Health Network, Bethlehem, Pennsylvania; b Department of Dermatology, St. Luke’s University Health Network, Bethlehem, Pennsylvania

**Keywords:** case study, clinical image, dermatology, nevus lipomatusus superficialis, photo, skin disease

A 67-year old man underwent a full body skin examination after avoiding contact with the health care system for 40 years. He noted a “large genital wart” of the buttocks (see Fig 1), diagnosed by a primary care provider when he was in his 20s. On examination, a 23 × 16 cm skin-colored, pedunculated, and multinodular growth was noted on his right buttock, extending from the gluteal cleft to the superior aspect of the thigh. The patient reported the lesion developed after a localized injury in childhood, had been slowly growing ever since, and had never received treatment. There was no hair, comedo-like lesions, or discharge associated.

**Figure:**
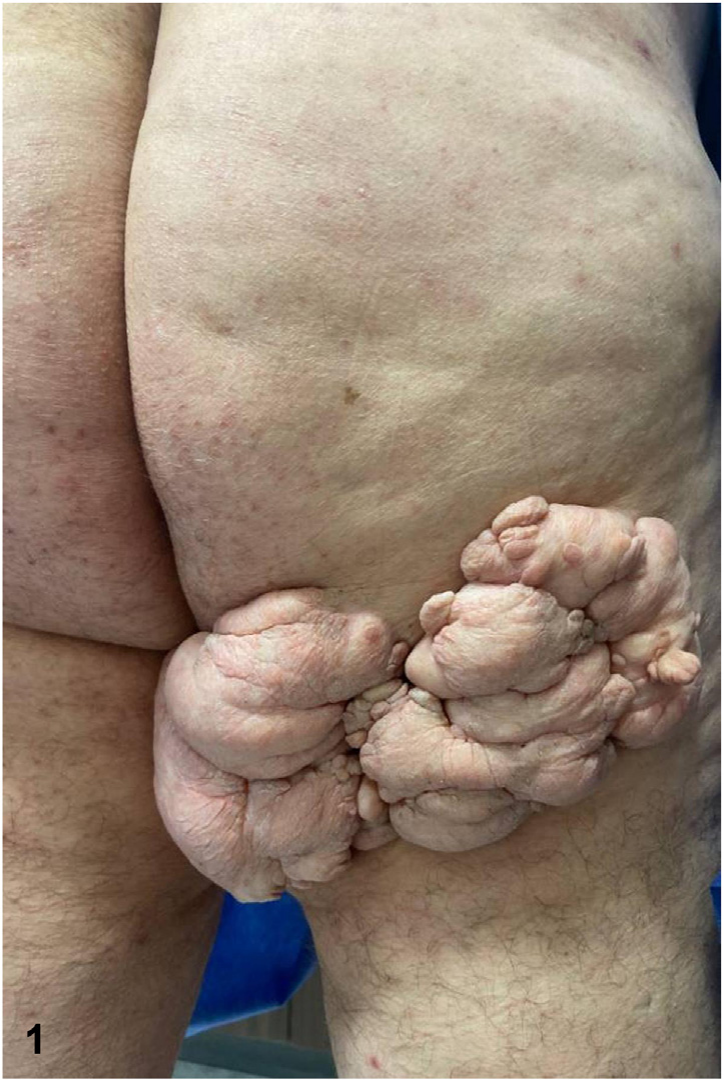

**Question 1: What is the most likely diagnosis?**

A. Nevus lipomatosus superficialis (NLS)
B. Acrochordon
C. Plexiform neurofibroma
D. Connective tissue nevus
E. Condyloma

**Answers:**

A. Nevus lipomatosus superficialis (NLS) – Correct. NLS can be categorized as classical or solitary. Because of the prototypical classical NLS gross appearance—skin-colored multinodular, cerebriform surface—in conjunction with pelvic involvement, unilateral linear arrangement, and presentation before age 20 years, our patient’s lesion would be classified as classical NLS with a precipitating injury to the involved area. Classical NLS can grow to be large over time. Rarely, in cases of prolonged duration, classical NLS can grow to the point of being characterized as giant (over 15 × 15 cm), often presenting with lumbar or buttocks involvement. Our case represents one of the largest ever reported. Although no specific associations have been proven, the classical type has been shown to co-occur with café-au-lait spots, cherry angiomas, localized achromoderma, overlying hair growth, comedo involvement, and angiokeratoma of Fordyce.^1^
B. Acrochordon – Incorrect. An acrochordon, or a “skin tag” is most often found in skinfolds, is smaller in size, and is not associated with an inciting event.
C. Plexiform neurofibroma – Incorrect. Neurofibromas often present as singular papules. Plexiform neurofibromas are usually congenital and undergo more growth in childhood. Our patient presents with a lesion occurring after trauma to the area that grew continuously throughout adulthood.^2^
D. Connective tissue nevus – Incorrect. Connective tissue nevus may present similarly to this lesion as single or multiple lesions on the trunk or extremities. They typically have “cobblestone,” “leather-grain,” or “peau d’orange” surfaces instead of the cerebriform lesion in this case. Occasionally they can be in a dermatomal distribution.^3^
E. Condyloma – Incorrect. Condylomas, what our patient was originally diagnosed with, can present similarly as coalesced lesions, are usually found in the oral or anogenital regions and are associated with human papillomavirus.

**Question 2: What is a most likely histologic feature of this lesion?**

A. Melanocyte invasion of surrounding tissue
B. Nonnative adipocytes in the reticular dermis
C. Hyperplasia of sebaceous glands
D. Infiltration of inflammatory cells in the dermis
E. Separation of the dermis and epidermis

**Answers:**

A. Melanocyte invasion of surrounding tissue – Incorrect. The presence of melanocytes in surrounding tissue would be indicative of melanoma, which would present clinically as a pigmented lesion with irregular borders.
B. Nonnative adipocytes in the reticular dermis – Correct. A punch biopsy and a shave biopsy were obtained from 2 different areas of the mass; both of which showed adipose tissue in the dermis consistent with NLS. NLS is a rare benign hamartoma that demonstrates mature adipocytes in the reticular dermis on histology. Although pathogenesis is currently unknown, it is thought that the nonnative adipocytes arise in the skin in utero during fetal lipogenesis.^4^
C. Hyperplasia of sebaceous glands – Incorrect. Hyperplasia of the sebaceous glands would be present in sebaceous hyperplasia, which is most often diagnosed in middle- to older-aged males, is mainly on the face, and presents as a yellow papule.
D. Infiltration of inflammatory cells in the dermis – Incorrect. Inflammatory cells in the dermis would not be seen in this case, as NLS does not have a foreign or inflammatory component.
E. Separation of the dermis and epidermis – Incorrect. Separation of the dermis and epidermis would be seen in bullous pemphigoid—our patient had no history of blistering or skin sloughing.

**Question 3: What is the next best step in management of this patient to optimize cosmetic outcomes?**

A. Systemic corticosteroids
B. Serial excisions or CO_2_ laser therapy
C. Radiation therapy
D. Topical antibiotics and/or antifungals
E. Systemic antibiotics and/or antifungals

**Answers:**

A. Systemic corticosteroids – Incorrect. Systemic corticosteroids have no reported effect on NLS. Rare cases in the literature have used topical corticosteroids to treat the symptoms of NLS, but our patient was asymptomatic.^5^
B. Serial excisions or CO_2_ laser therapy – Correct. Because the lesion has remained asymptomatic, and without visceral involvement, obstruction, or malignant transformation, removal at this point would be considered cosmetic and likely necessitate serial excisions or CO_2_ laser, with the possibility of recurrence. This patient was then referred to plastic surgery for evaluation of the next steps to optimize cosmesis and comfort. When left untreated, NLS can grow into mammoth lesions that present therapeutic challenges and lead to significant emotional distress. Vigilant surveillance and early intervention, coupled with proper patient informing, can optimize patient physical and psychological outcomes.^5^
C. Radiation therapy – Incorrect. Radiation therapy would most likely damage the surrounding tissue and could leave the patient susceptible to future complications.^5^
D. Topical antibiotics and/or antifungals – Incorrect. Antibiotics and antifungals are inappropriate as there is no infectious component in this case.
E. Systemic antibiotics and/or antifungals – Incorrect. Antibiotics and antifungals are inappropriate as there is no infectious component in this case.

## Conflicts of interest

None disclosed.
